# Guilt, shame, and fear in relation to posttraumatic growth through psychological flexibility among adults reporting dating violence exposure

**DOI:** 10.3389/fpsyg.2026.1865973

**Published:** 2026-07-30

**Authors:** Leyla Aziz, Serap Çakıcı, Ahmet Sapanci

**Affiliations:** 1 Faculty of Social Sciences and Humanities, Department of Psychology, İstanbul Arel University, İstanbul, Türkiye; 2 Faculty of Social Sciences and Humanities, Department of Psychology, İstanbul Rumeli University, İstanbul, Türkiye; 3 Department of Psychological Counseling and Guidance, Düzce University, Düzce, Türkiye

**Keywords:** dating violence, fear, guilt, posttraumatic growth, psychological flexibility, shame

## Abstract

Dating violence can be experienced as a form of relational trauma, yet comparatively little is known about whether guilt, shame, and fear show differentiated indirect associations with posttraumatic growth when global psychological flexibility is specified as an intervening construct among adults reporting dating violence exposure. This cross-sectional study tested a hypothesized structural equation model in 396 adults (*M* = 32.15, SD = 9.33) who responded affirmatively to a dichotomous screening item concerning prior dating violence. The item was used solely to determine eligibility, and dating violence was not modeled as an explanatory variable. Shame (*β* = −0.170, *p* = 0.007) and fear (*β* = −0.423, *p* < 0.001) were negatively associated with psychological flexibility, whereas guilt was positively associated with psychological flexibility (*β* = 0.158, *p* = 0.009). Psychological flexibility, assessed as a broad construct, was positively associated with posttraumatic growth (*β* = 0.613, *p* < 0.001). Bootstrapped analyses with 5,000 resamples showed significant indirect associations for fear (*β* = −0.259, 95% bias-corrected CI [−0.343, −0.187]), shame (*β* = −0.105, 95% bias-corrected CI [−0.188, −0.030]), and guilt (*β* = 0.097, 95% bias-corrected CI [0.021, 0.180]). These findings indicate differentiated cross-sectional indirect associations between emotional experiences and posttraumatic growth in a model including psychological flexibility. Because all variables were measured concurrently and dating violence was used only as an eligibility criterion, the results do not establish temporal precedence, causal mediation, or variation according to the severity, frequency, duration, or type of violence exposure.

## Introduction

Intimate partner violence (IPV) refers to behaviors perpetrated by a current or former partner that result in physical, sexual, or psychological harm, including physical assault, sexual coercion, and controlling behaviors (World Health Organization, 2024). IPV extends beyond isolated acts and may reflect persistent patterns of power, control, and coercion within intimate relationships (Johnson, 2008; Walker, 1979).

Violence against women remains an important public health and social concern in Türkiye. According to the Turkish Statistical Institute, 2025, lifetime prevalence estimates included psychological violence (28.2%), economic violence (18.3%), physical violence (12.8%), stalking (10.9%), digital violence (8.3%), and sexual violence (5.4%; Turkish Statistical Institute, 2025). Because these estimates include multiple perpetrator categories, they should not be interpreted as dating-violence- or IPV-specific prevalence rates. Nevertheless, they illustrate the broader national burden of violence against women and provide context for research on violence occurring within romantic relationships.

The present study focuses on dating violence within nonmarital adult romantic relationships. Although dating violence shares core features with IPV, it constitutes a specific relational context in which emotional, interpersonal, and developmental processes may shape individuals’ responses to violence. Exposure to partner violence has been associated with depression, anxiety, posttraumatic stress symptoms, and suicidality (Lagdon et al., 2014; White et al., 2024). Self-blame and shame have also been identified as relevant trauma-related responses in interpersonal victimization contexts (Feiring and Taska, 2005). Decisions to remain in or leave abusive relationships may also be influenced by perceived investment, available alternatives, commitment, dependency, and relational constraints (Rhatigan and Axsom, 2006).

### Relational, gendered, and power-based context of dating violence

Dating violence should not be reduced to an individual emotional-processing problem. Feminist and relational-trauma perspectives emphasize that emotional responses to partner violence develop within interpersonal and social contexts characterized by coercive control, unequal power relations, dependency, victim-blaming, stigma, and gendered expectations (Herman, 1992; Stark, 2007). From this perspective, shame following partner violence may reflect not only an internal negative evaluation of the self but also the internalization of blame, social devaluation, and messages communicated within an abusive relationship or the broader social environment.

Betrayal trauma theory further suggests that violence perpetrated by a trusted or emotionally significant person may disrupt assumptions regarding safety, trust, and relational security (Freyd, 1996). Relational entrapment and coercive control may constrain perceived alternatives and complicate individuals’ efforts to interpret or disengage from an abusive relationship (Rhatigan and Axsom, 2006; Stark, 2007). These perspectives provide an important contextual framework for understanding guilt, shame, and fear. However, coercive control, gendered power, victim-blaming, betrayal, and relational entrapment were not directly measured in the present study and are therefore not treated as empirically tested explanatory variables.

Importantly, traumatic experiences do not invariably lead to psychopathology. Individuals may report positive psychological changes following adversity, conceptualized as posttraumatic growth (PTG) (Tedeschi and Calhoun, 2004). PTG involves multidimensional changes, including enhanced self-perception, strengthened interpersonal relationships, recognition of new possibilities, and a restructured sense of meaning in life (Tedeschi et al., 2018).

Contemporary PTG scholarship conceptualizes growth as involving interconnected cognitive, emotional, relational, and meaning-making processes rather than as an exclusively cognitive outcome. Organismic Valuing Theory proposes that growth may emerge when individuals are able to process trauma-related experiences in ways that support the reconstruction of goals, identity, and personally meaningful directions (Joseph and Linley, 2005). Similarly, the Meaning Making Model emphasizes the processes through which individuals attempt to reconcile the appraised meaning of an adverse event with broader beliefs, goals, and global meaning systems (Park, 2010). Research has also examined emotional regulation, emotional disclosure, experiential avoidance, self-compassion, psychological flexibility, coping, and social support as processes associated with PTG (Joseph and Linley, 2005; Park, 2010; Prati and Pietrantoni, 2009; Tedeschi et al., 2018). Accordingly, the present study does not assume that emotional processes have been generally neglected in the PTG literature.

Emerging research also questions whether self-reported PTG consistently reflects enduring transformation, suggesting that it may sometimes represent positive reinterpretation, coping-related appraisal, or defensive reconstruction (Jayawickreme et al., 2021). The distinction between constructive adaptation and perceived or illusory growth highlights the importance of examining the regulatory processes associated with reports of positive posttraumatic change. The present cross-sectional study cannot determine whether reported PTG reflects objectively verifiable or enduring transformation; it examines only the associations among self-reported psychological variables.

### Posttraumatic growth in the context of interpersonal and partner violence

PTG has previously been examined in the context of interpersonal violence, IPV, and relational trauma. Existing studies have considered meaning reconstruction, resilience, identity transformation, social support, barriers to growth, and the coexistence of distress and positive perceived change following abusive relationships. Systematic reviews have synthesized evidence concerning PTG after interpersonal violence (Elderton et al., 2017), while qualitative studies have documented complex pathways of recovery and perceived growth among women who had experienced IPV (Bryngeirsdottir and Halldorsdottir, 2022a, 2022b).

Trauma-specific accounts of posttraumatic growth after intimate partner violence further portray recovery as a multidimensional and potentially nonlinear process shaped by prior trauma, the consequences of violence, facilitating and hindering conditions, and lingering effects that may coexist with perceived growth (Bryngeirsdottir et al., 2022). This perspective supports treating psychological flexibility as one potentially relevant regulatory correlate within a broader relational and ecological recovery context rather than as a sufficient or exclusive explanation of posttraumatic growth.

This literature indicates that positive change following partner violence does not arise automatically from exposure. Rather, it may be shaped by the relational context of the violence, opportunities for safety and social support, reconstruction of identity and meaning, and the manner in which trauma-related emotions are processed.

### Self-conscious emotions and threat-based emotional systems

Guilt, shame, and fear are theoretically relevant but functionally distinct emotional systems that may be associated with adaptation among adults reporting dating violence. In this study, guilt, shame, and fear are analyzed within motivational frameworks grounded in self-evaluation and threat perception (Tangney et al., 2007). Although guilt and shame are both self-conscious emotions, guilt is organized around negative evaluations of specific behaviors (“I did something wrong”), whereas shame involves a more global negative evaluation of the self (“I am inadequate”) (Lewis, 2008).

This distinction is critical to understanding their functional implications. Guilt, which focuses on behavior, is more likely to activate reparative processes; individuals may evaluate consequences, assume responsibility, and attempt relationship repair (Baumeister et al., 1994; Leith and Baumeister, 1998). Accordingly, guilt can play an adaptive role by facilitating empathy and constructive cognitive processing (Tangney et al., 2007). Conversely, shame is associated with self-focused negative evaluation, withdrawal, concealment, and avoidance. These tendencies may undermine interpersonal functioning and impede open processing of emotional experiences (Morrison, 1989; Tangney et al., 2007).

In the context of partner violence, however, shame should not be interpreted solely as an internally generated cognitive appraisal. Shame may also be socially and relationally produced through coercive control, degradation, victim-blaming, perceived stigma, and gendered expectations concerning responsibility for relationship maintenance (Herman, 1992; Stark, 2007). Thus, a person may experience shame even when responsibility for the violence lies with the perpetrator. Because these relational and social mechanisms were not directly measured, the present study cannot determine the sources or functions of participants’ shame responses.

Fear, in contrast to self-conscious emotions, is a fundamental emotional system grounded in threat detection. This system alerts the organism to danger and activates protective responses (Eysenck and Derakshan, 2011; LeDoux, 2000). However, when fear becomes persistent, it may narrow cognitive and emotional functioning by amplifying threat-focused attention and reinforcing avoidance. In traumatic contexts, this constriction may undermine perceived safety and hinder the reflective and meaning-making processes associated with adaptive change.

### Processing of emotional experiences

The psychological implications of trauma-related emotions may vary as a function of how these emotions are processed. Mechanisms such as repetitive rumination, experiential avoidance, and cognitive constriction can promote rigid, threat-focused processing that hinders adaptation. In contrast, cognitive reappraisal, emotional engagement, social disclosure, and meaning-making may support more flexible responses to adversity (Gross, 2015; Joseph and Linley, 2005; Park, 2010).

Meta-analytic findings indicate that shame and guilt are associated with posttraumatic stress, but the strength and direction of these associations vary according to the form of the emotion and the manner in which it is processed or measured (Shi et al., 2021). Guilt, when characterized by rumination or self-punishment, may increase distress; when focused on specific behavior and integrated with responsibility-taking and meaning-making, it may support reparative action (Tangney et al., 2007; Wang et al., 2018). Conversely, shame and persistent fear may be associated with withdrawal, avoidance, and threat-based constriction (Feiring and Taska, 2005; Tangney et al., 2007).

### The role of psychological flexibility in emotional processing

Psychological flexibility (PF) refers to the capacity to remain open to challenging internal experiences, maintain contact with the present context, and engage in behavior consistent with personally meaningful values (Hayes et al., 1999; Hayes et al., 2011). Rather than requiring the elimination or alteration of difficult thoughts and emotions, PF concerns the manner in which individuals relate and respond to them. Lower PF is commonly characterized by experiential avoidance, cognitive fusion, and behavioral constriction, whereas greater PF is associated with openness, contextual responsiveness, and values-consistent action (Kashdan and Rottenberg, 2010).

By reducing rigid avoidance and supporting value-oriented behavior, psychological flexibility has been associated with a range of mental-health and adaptive-functioning outcomes (Gloster et al., 2017). Emerging evidence also links psychological flexibility with PTG (Sarıkoç and Uğur, 2025), suggesting that it may be relevant to how individuals engage with trauma-related thoughts and emotions.

Nevertheless, the role of psychological flexibility should not be interpreted as exclusive or determinative. PTG may also be associated with social support, safety, meaning-making, material resources, identity reconstruction, and the broader relational context in which recovery occurs. Moreover, because the present study uses a broad psychological flexibility score, it cannot determine whether any particular ACT process—such as cognitive defusion, acceptance, self-as-context, present-moment awareness, or values-based action—is primarily responsible for the observed associations.

Accordingly, guilt, shame, and fear may show different associations with PF. Persistent fear and global shame may be associated with experiential avoidance and restricted behavioral repertoires, whereas behavior-focused guilt may, under some conditions, be associated with responsibility-taking and value-consistent action. These propositions concern associations among broad constructs and should not be interpreted as direct tests of process-specific ACT mechanisms.

### Emotional experiences and posttraumatic growth

Posttraumatic growth involves perceived positive psychological changes following highly challenging life events (Tedeschi et al., 2018; Wu et al., 2019). The term “perceived” is important because self-reported PTG does not necessarily establish enduring behavioral or personality transformation. PTG may develop alongside continuing distress and may involve both cognitive reconstruction and emotional engagement.

Debates persist regarding whether reported PTG reflects genuine transformation, adaptive meaning reconstruction, coping-related positive appraisal, or defensive cognitive reframing (Jayawickreme et al., 2021). This debate highlights the importance of examining the psychological processes associated with PTG while avoiding the assumption that higher scores necessarily represent objectively verified change. While trauma may disrupt core assumptions and initiate cognitive restructuring (Janoff-Bulman, 1992; Tedeschi and Calhoun, 2004), this process may also be shaped by emotional regulation, relational safety, social support, and opportunities for meaning reconstruction.

### Recent empirical research on psychological flexibility, emotional processes, and posttraumatic growth

Recent empirical studies have examined psychological flexibility and related regulatory processes as correlates or predictors of posttraumatic growth across several trauma contexts. In a short-term longitudinal study conducted during the COVID-19 pandemic, Landi et al. (2022) found that psychological flexibility was associated with higher subsequent posttraumatic growth, particularly among individuals reporting elevated posttraumatic stress. Present-moment awareness, cognitive defusion, values, and committed action were among the flexibility processes associated with subsequent growth in this high-distress group. In a separate one-year follow-up study, Bruno et al. (2024) found that psychological inflexibility was associated with lower subsequent posttraumatic growth, whereas problem-oriented coping was positively associated with growth. Similarly, Türk (2024) reported that psychological flexibility moderated the association between COVID-19-related traumatic stress and posttraumatic growth, while resilience statistically mediated this association. Collectively, these findings indicate that the relevance of psychological flexibility to posttraumatic growth has already received empirical support and is not, by itself, the novel contribution of the present study.

Recent cross-sectional mediation studies have further situated psychological flexibility within broader multivariable models of posttraumatic adjustment. In a sample of 298 Indian young adults reporting adverse childhood experiences, Sachdeva and Shukla (2024) found that psychological flexibility and self-compassion statistically mediated the association between adverse childhood experiences and posttraumatic growth. In a clinical sample of patients with breast or gynecological cancer, Akcan et al. (2024) reported a statistically significant indirect association between psychological flexibility and posttraumatic growth through active coping. These studies suggest that psychological flexibility is more appropriately understood as one regulatory resource embedded within broader networks of coping, resilience, self-compassion, and trauma-related distress, rather than as a sufficient or exclusive explanation of posttraumatic growth.

Recent empirical research also demonstrates that self-conscious emotions may have differentiated and context-dependent associations with posttraumatic outcomes. In a three-wave study of trauma-exposed Chinese university students, Li et al. (2025) examined trauma-related shame and guilt as explanatory variables linking compassionate and uncompassionate self-responding with posttraumatic stress symptoms and posttraumatic growth. Trauma-related guilt, but not trauma-related shame, accounted for some of the longitudinal associations with posttraumatic growth. This pattern cautions against treating guilt and shame as interchangeable forms of negative affect. It also highlights an important measurement distinction: whereas Li et al. assessed emotions specifically related to traumatic experiences, the present study assessed general guilt and shame and therefore cannot determine whether participants’ emotional scores originated from, or were specifically directed toward, their dating-violence experiences.

Empirical studies conducted specifically with intimate partner violence survivors further position posttraumatic growth within temporal, identity-related, relational, and sociocultural recovery processes. In an 18-month, three-wave longitudinal study involving 217 women who had experienced intimate partner violence, Bakaitytė et al. (2022) found that changes in posttraumatic growth varied according to the time elapsed since violence exposure. Initial levels of growth were also positively associated with event centrality and identity exploration, although these variables did not predict subsequent change in growth. In a structural equation model involving 261 Jewish Ultra-Orthodox intimate partner violence survivors, Szyfer Lipinsky et al. (2025) examined a broader recovery system encompassing cultural and religious norms, trauma-related cognitions, recovery actions, faith-based responses, help-seeking, well-being, and psychopathology. These findings reinforce that recovery following partner violence cannot be reduced to a single intrapersonal regulatory process. Psychological flexibility should instead be considered one potentially relevant correlate within a wider relational, temporal, cultural, and ecological recovery context.

Against this empirical background, the unresolved question is not whether psychological flexibility is associated with posttraumatic growth. Rather, comparatively less is known about whether guilt, shame, and fear—assessed simultaneously as broad, non-trauma-specific constructs—show unique associations with psychological flexibility after their shared variance is taken into account and corresponding statistical indirect associations with self-reported posttraumatic growth through psychological flexibility in an exposure-selected sample of adults reporting dating violence. The latent-variable model permits these mutually adjusted associations to be estimated but does not establish emotion-specific mechanisms, temporal precedence, or causal recovery pathways.

### Cultural considerations

Cultural contexts may influence the meaning, expression, and regulation of self-conscious and threat-related emotions (Markus and Kitayama, 1991; Mesquita and Walker, 2003). However, the present study did not assess collectivism, relational self-construal, cultural norms, perceived social expectations, or culturally specific meanings of guilt, shame, fear, and PTG. Culture-specific explanations therefore cannot be derived from the present data and are not used to account for the observed structural associations.

The fact that the study was conducted in Türkiye identifies the sociogeographic context of the sample but does not establish that the findings were produced by Turkish collectivism or relational cultural orientations. Directly measured cultural variables and cross-cultural or measurement-invariance designs would be required to evaluate such explanations. Cultural influences are therefore treated only as a potentially relevant direction for future research.

Within the proposed theoretical framework, guilt, shame, and fear are conceptualized as functionally distinct emotional experiences whose associations with posttraumatic growth may vary according to how individuals relate to them. Psychological flexibility was specified as a theoretically relevant linking variable in these associations. This specification represents a parsimonious correlational model rather than a comprehensive ACT, feminist-trauma, relational-trauma, or culturally specific explanatory model.

Accordingly, the present study is positioned as a modest, context-specific extension of existing correlational process research rather than as a new explanatory model of posttraumatic growth or recovery. Its incremental contribution lies in jointly estimating, within a single latent-variable model, the mutually adjusted associations of guilt, shame, and fear—assessed as broad, non-trauma-specific constructs—with psychological flexibility and their corresponding statistical indirect associations with self-reported posttraumatic growth through psychological flexibility in a predominantly female sample selected on the basis of reported dating-violence exposure. The study does not estimate the effects of dating violence itself or test trauma-specific emotional responses, specific ACT processes, temporal ordering, or causal recovery pathways.

The hypothesized model therefore specified negative indirect associations of shame and fear with posttraumatic growth through psychological flexibility and a positive indirect association of guilt. Because the design was cross-sectional, this ordering represents a theoretically specified correlational structure rather than temporal precedence, causal mediation, or evidence of process-specific ACT or culturally specific mechanisms. Dating-violence exposure served only as the sample-defining criterion and was not included as a structural variable.

*H1*: Shame will show a negative indirect association with posttraumatic growth through psychological flexibility.

*H2*: Fear will show a negative indirect association with posttraumatic growth through psychological flexibility.

*H3*: Guilt will show a positive indirect association with posttraumatic growth through psychological flexibility.

## Method

### Research design

This study employed a quantitative, cross-sectional, and correlational design. Structural equation modeling (SEM) was used to estimate direct and indirect associations among the variables. Consistent with the theoretical framework, guilt, shame, and fear were specified as exogenous latent variables, whereas psychological flexibility and posttraumatic growth (PTG) were modeled as endogenous latent variables.

### Participants

Data were initially obtained from 401 eligible adults. During data screening, five cases were removed because they had standardized scores exceeding the prespecified univariate outlier criterion of |z| > 3.30 on at least one study variable. The final analytical sample therefore comprised 396 adults (M = 32.15, SD = 9.33, range = 18–60). All reported analyses were conducted using this final sample.

All participants responded affirmatively to a dichotomous yes/no item asking whether they had experienced dating violence. Participants were recruited using nonprobability convenience sampling. Eligibility criteria were (a) being 18 years of age or older, (b) providing an affirmative response to the dating violence screening item, and (c) providing informed consent. Because affirmative endorsement was an eligibility criterion, dating-violence exposure showed no variation in the analytical sample and was not included as a variable in the structural model.

As detailed in Table 1, the sample was predominantly female. Of the 396 participants, 335 selected the “Female” response option (84.6%), 59 selected the “Male” response option (14.9%), and two selected the “Other” response option (0.5%). Most participants held a bachelor’s degree (56.3%) and were employed (69.2%). Given the use of convenience sampling and the substantial gender imbalance in the sample, the findings should not be considered representative of the broader population of adults who report having experienced dating violence.

**Table 1:** Demographic characteristics of the participants.

Variable	Category	*n*	%
Gender	Female	335	84.6
	Male	59	14.9
	Other	2	0.5
Marital Status	Married	144	36.4
	Single	220	55.6
	Divorced/widowed	31	7.8
	Cohabiting	1	0.3
Education level	Literate (no formal schooling)	2	0.5
	Primary school	4	1.0
	Middle school	5	1.3
	High school	65	16.4
	Bachelor’s degree	223	56.3
	Graduate degree	97	24.5
Employment status	Employed	274	69.2
	Not employed	120	30.3
Current romantic relationship	Yes	187	47.2
	No	206	52.0

Percentages were calculated using the total analytical sample (*N* = 396). Two participants did not report their employment status, and three did not report their current romantic relationship status. Percentages may not total 100 due to missing responses and rounding.

### Measures

### Demographic information form

A researcher-developed demographic information form assessed age, gender, marital status, educational level, employment status, and current and previous romantic relationship status. The form also included a dichotomous yes/no item asking whether the participant had experienced dating violence. This item was used solely to determine eligibility and did not assess the severity, frequency, duration, recency, or specific type of dating violence. It was not intended to operationalize dating violence as a multidimensional study construct and was not used as a predictor, outcome, moderator, mediator, or explanatory variable in the structural model. Rather, an affirmative response served only to define the relational context of the analytical sample. Accordingly, the study did not estimate the effects of dating violence itself or examine whether the observed psychological associations varied according to violence characteristics.

### Guilt–shame scale (GSS)

The Guilt and Shame Scale (Şahin and Şahin, 1992) assesses experiences of guilt and shame through 24 items, comprising two 12-item subscales. Items are rated on a 5-point Likert scale (1 = not at all appropriate, 5 = completely appropriate), with higher scores indicating higher levels of guilt or shame. The scale was selected primarily because it has been widely used in the Turkish literature and provides separate scores for guilt and shame. The original internal consistency coefficients were *α* = 0.81 for guilt and *α* = 0.80 for shame. In the present study, Cronbach’s alpha coefficients were 0.80 and 0.78, respectively. Consistent with their theoretical distinction, guilt and shame were modeled as separate exogenous latent variables. The guilt subscale provides a general assessment of guilt and does not distinguish among adaptive, maladaptive, trauma-related, or victimization-specific forms of guilt or self-blame. Accordingly, guilt scores were interpreted as reflecting general guilt rather than a specific guilt subtype.

### Fear and helplessness scale

The Fear and Helplessness Scale (Salcioglu et al., 2017) measures expectancy-based fear and perceived loss of control through 10 items rated on a 5-point Likert scale (1 = not at all true, 5 = very true). Consistent with the theoretical framework of the present study, only the 6-item fear subscale, which assesses ongoing danger perception and fear responses, was used. The original subscale reliability was *α* = 0.81, and Cronbach’s alpha was 0.83 in the present study.

### Psychological flexibility scale

The Psychological Flexibility Scale (Francis et al., 2016; Karakuş and Akbay, 2020) consists of 28 items representing five dimensions: values-based action, present-moment awareness, acceptance, self-as-context, and cognitive defusion. Items are rated on a 7-point Likert scale (1 = strongly disagree, 7 = strongly agree), with higher total scores indicating greater psychological flexibility after reverse-scored items are recoded. The validation study of the adapted version reported a total-scale reliability coefficient of α = 0.79 (Karakuş and Akbay, 2020). In the present study, Cronbach’s alpha coefficients were 0.89 for values-based action, 0.85 for present-moment awareness, 0.74 for acceptance, 0.68 for self-as-context, and 0.55 for cognitive defusion. The total-scale internal consistency coefficient was α = 0.82.

### Posttraumatic growth inventory (PTGI-X)

The Posttraumatic Growth Inventory–Expanded (Tedeschi et al., 2017) is a 25-item revised form of the original inventory (Tedeschi and Calhoun, 1996), assessing personal strength, new possibilities, relating to others, appreciation of life, and spiritual–existential change. Items are rated on a 6-point Likert scale (0 = not at all, 5 = to a very great degree). In the present study, internal consistency coefficients for the subscales ranged from 0.70 to 0.84 (α_total = 0.94). Posttraumatic growth was modeled as a multidimensional latent construct using its original subscale indicators to preserve the conceptual distinctiveness of its dimensions.

### Ethics and procedure

The authors assert that all procedures contributing to this work complied with the ethical standards of the relevant national and institutional committees on human experimentation and with the Declaration of Helsinki of 1975, as revised in 2013. All procedures involving human participants were approved by the Istanbul Arel University Ethics Committee (approval date: December 21, 2021; approval number: E-69396709-050.01.04-199270). Informed consent was obtained from all participants, who were informed of the study aims and their right to withdraw at any time without penalty. Data were collected anonymously, and no identifying information was recorded. Data collection was conducted online and face-to-face between January and December 2022.

### Statistical analysis

Prior to the primary analyses, the dataset was screened for missing values, univariate and multivariate outliers, and distributional assumptions. The initial dataset consisted of 401 participants. Five cases with standardized scores exceeding the univariate outlier criterion of |z| > 3.30 were removed before the reported analyses, resulting in a final analytical sample of 396 participants. One case exceeded the prespecified Mahalanobis distance threshold for multivariate outliers. However, Cook’s distances were uniformly low (maximum = 0.087), indicating no undue influence; therefore, the case was retained.

Missingness was examined separately for demographic variables and scale items. Missing demographic responses were limited to employment status (*n* = 2) and current romantic relationship status (*n* = 3). No missing observations were present in the scale items or observed indicators included in the measurement and structural models. Therefore, no imputation, deletion due to missing scale data, or model-based missing-data procedure was required. All descriptive, correlational, confirmatory factor, and structural equation modeling analyses were conducted using the final analytical sample of *N* = 396. Skewness and kurtosis values were within acceptable ranges, supporting approximate univariate normality (Hair et al., 2019; Tabachnick and Fidell, 2013).

Dating violence status was not included in the structural model because affirmative endorsement of the screening item was an eligibility criterion and therefore showed no between-participant variability. Because no information was collected on the severity, frequency, duration, recency, or type of violence exposure, the analyses did not estimate the effects of dating violence or examine whether the modeled psychological associations varied according to violence characteristics.

Prior to model estimation, relationships among the variables were examined using two-tailed Pearson correlation analyses (*α* = 0.05). Intercorrelations among the exogenous variables were inspected for potential multicollinearity. Discriminant validity was assessed using heterotrait–monotrait (HTMT) ratios, with values below 0.85 indicating adequate discriminant validity (Henseler et al., 2015). Structural equation modeling (SEM) was conducted using maximum likelihood estimation in IBM SPSS Amos Version 25. The measurement model was examined using confirmatory factor analysis (CFA). Model fit was assessed using the *χ*^2^ statistic, *χ*^2^/df ratio, comparative fit index (CFI), Tucker–Lewis index (TLI), and root mean square error of approximation (RMSEA). Values of *χ*^2^/df < 3, CFI and TLI ≥ 0.90, and RMSEA ≤ 0.08 were considered indicative of acceptable model fit.

Because the psychometric performance of the Guilt–Shame Scale has not been established specifically in dating violence or intimate partner violence samples, its proposed two-factor structure was additionally examined at the item level in the present sample before parcel formation. A CFA was specified in which the 12 guilt items loaded on the guilt factor and the 12 shame items loaded on the shame factor, with the two latent factors allowed to correlate. Model fit was evaluated using *χ*^2^/*df*, CFI, TLI, IFI, GFI, RMSEA with its 90% confidence interval, and PCLOSE. The results of this item-level CFA are reported in the measurement-model subsection.

Parceling was used to reduce indicator-level model complexity while retaining latent-variable estimation. However, parceling was not treated as evidence that the underlying constructs were strictly unidimensional. Because parceling may conceal local misfit, poorly performing items, or facet-level heterogeneity, item-level, subscale-indicator, and nonparceled sensitivity analyses were conducted to evaluate the robustness and limitations of the parcel-based specification. Guilt and shame were each represented by three parcels and fear by two parcels using the item-to-construct balance approach (Little et al., 2002; Matsunaga, 2008). Psychological flexibility was represented by three item parcels constructed using a serpentine allocation procedure based on corrected item–total correlations. All reverse-scored items were recoded before parcel formation, and the direction of the subscale and total scores was verified. The 28 items were ranked in descending order according to the corrected item–total correlations obtained from the total-scale reliability analysis and allocated sequentially across the three parcels using a repeating 1–2–3–3–2–1 sequence.

PF1 consisted of Items 1, 2, 7, 8, 11, 16, 17, 25, and 26; PF2 consisted of Items 5, 10, 12, 20, 21, 22, 24, 27, and 28; and PF3 consisted of Items 3, 4, 6, 9, 13, 14, 15, 18, 19, and 23. PF1 and PF2 each contained 9 items, whereas PF3 contained 10 items. Parcel scores were calculated by summing the constituent item scores. The serpentine allocation procedure was used to distribute items with relatively higher and lower corrected item–total correlations across the three indicators; it was not intended to ensure identical representation of all five psychological flexibility subscales within each parcel.

Posttraumatic growth was represented by its five original subscale scores, which served as indicators of a multidimensional latent construct, thereby preserving the conceptual distinctiveness of its dimensions.

To examine whether the parcel-based representation concealed subscale-level measurement heterogeneity, a sensitivity model was estimated in which the five original psychological flexibility subscale scores—values-based action, present-moment awareness, acceptance, self-as-context, and cognitive defusion—were used as indicators of the psychological flexibility construct. Because the parcel-based and subscale-indicator specifications used different observed indicators and were not nested, their model-fit indices and structural coefficients were compared descriptively rather than through a formal chi-square difference test.

To examine whether the substantive structural findings depended on item parceling, the hypothesized indirect-effects model was also re-estimated using the observed total scores of shame, guilt, fear, psychological flexibility, and posttraumatic growth. The same indirect-only structural specification was retained, with shame, guilt, and fear specified as correlated exogenous variables. Because the parcel-based, subscale-indicator, and observed-score models used different indicators and were not nested, their fit indices and structural estimates were compared descriptively rather than through formal chi-square difference testing.

Convergent validity was assessed using average variance extracted (AVE) and composite reliability (CR), whereas discriminant validity was evaluated using the Fornell–Larcker criterion and HTMT ratios. Potential common method variance was evaluated using Harman’s single-factor test, a single-factor CFA, and an unmeasured latent method construct (ULMC) approach. These procedures were treated as *post hoc* diagnostic analyses rather than as definitive tests capable of ruling out common method bias. The ULMC model was compared with the original measurement model to examine whether the inclusion of a general latent method factor meaningfully altered model fit or parameter estimates. Within the structural model, indirect-only and partial structural specifications were compared using chi-square difference tests (Δ*χ*^2^) and the Akaike information criterion (AIC). Indirect effects across the structural specifications were evaluated using 5,000 bootstrap resamples and 95% bias-corrected confidence intervals.

## Results

All results reported below are based on the cleaned analytical sample of 396 participants. No missing observations were present in the scale items or observed indicators included in the analyses. All 396 participants included in the analytical sample responded affirmatively to the dating violence screening item. Because this variable was invariant within the sample, no correlations or structural paths involving dating violence status were estimated. This section presents the descriptive statistics and correlations among the study variables, followed by the results of the measurement and structural models.

### Descriptive statistics and correlations

Preliminary analyses examined the distributional properties of the variables and their interrelationships. Descriptive statistics and Pearson correlation coefficients are presented in Table 2.

**Table 2:** Descriptive statistics and Pearson correlations among study variables.

Variable	M	SD	1	2	3	4	5
1. Shame	43.06	8.40	-				
2. Guilt	51.43	6.67	0.295**	-			
3. Fear	21.31	8.72	0.254**	−0.119*	-		
4. Psychological flexibility	92.49	17.39	−0.225**	0.139**	−0.437**	-	
5. Posttraumatic growth	78.67	23.64	−0.136**	0.136**	−0.322**	0.550**	-

*p < 0.05, **p < 0.01.

Results demonstrated association patterns consistent with the theoretical framework. Shame was positively correlated with guilt (*r* = 0.295, *p* < 0.01). Fear was negatively associated with both psychological flexibility (*r* = −0.437, *p* < 0.01) and posttraumatic growth (*r* = −0.322, *p* < 0.01). Conversely, psychological flexibility showed a strong positive correlation with posttraumatic growth (*r* = 0.550, *p* < 0.01), representing a moderate-to-large effect size (Cohen, 1988). The association between shame and posttraumatic growth remained relatively weak (*r* = −0.136). Overall, the bivariate associations were generally consistent with the directions specified in the structural model.

### Measurement model

Before evaluating the overall measurement model, the proposed two-factor structure of the Guilt–Shame Scale was examined at the item level. The item-level two-factor CFA yielded *χ*^2^(242) = 520.66, *p* < 0.001, *χ*^2^/df = 2.15, RMSEA = 0.054, 90% CI [0.048, 0.060], PCLOSE = 0.148, GFI = 0.901, CFI = 0.867, TLI = 0.848, and IFI = 0.869. Although *χ*^2^/df, RMSEA, and GFI indicated acceptable fit, the incremental fit indices remained below conventional thresholds. Accordingly, the proposed two-factor structure received only partial support in the present sample. These findings indicate that the guilt and shame results should be interpreted with additional psychometric caution and should not be regarded as evidence that the scale has been fully validated in dating-violence or intimate partner violence populations.

The overall measurement model demonstrated good fit to the data: *χ*^2^(94) = 223.15, *χ*^2^/df = 2.37, CFI = 0.963, TLI = 0.952, RMSEA = 0.059, 90% CI [0.049, 0.069]. Standardized factor loadings ranged from 0.639 to 0.981 and were all statistically significant. These results indicated good global fit for the parcel-based measurement model, although the subsequent subscale-indicator sensitivity analysis revealed meaningful local measurement heterogeneity.

### Convergent validity and reliability

Convergent validity and reliability were assessed using Average Variance Extracted (AVE) and Composite Reliability (CR). As presented in Table 3, AVE values exceeded 0.50 and CR values exceeded 0.70 for all constructs. These results indicate satisfactory internal consistency and provide further support for the convergent validity of the measurement model.

**Table 3:** Convergent validity and composite reliability indicators.

Variable	CR	AVE	√AVE
Shame	0.82	0.60	0.77
Guilt	0.82	0.61	0.78
Fear	0.91	0.83	0.91
Psychological flexibility	0.84	0.64	0.80
Posttraumatic growth	0.90	0.66	0.81

CR, composite reliability; AVE, average variance extracted; √AVE, square root of the average variance extracted.

### Discriminant validity

Discriminant validity was assessed using the Fornell–Larcker criterion and the Heterotrait–Monotrait (HTMT) ratio. As shown in Table 4, the square root of the Average Variance Extracted (AVE) for each construct exceeded the absolute value of its correlations with the other constructs, supporting discriminant validity according to the Fornell–Larcker criterion.

**Table 4:** Discriminant validity results based on the Fornell–Larcker criterion.

Variable	1	2	3	4	5
1. Shame	0.77				
2. Guilt	0.334***	0.78			
3. Fear	0.267***	−0.155**	0.91		
4. Psychological flexibility	−0.226***	0.159**	−0.484***	0.80	
5. Posttraumatic growth	−0.155**	0.143*	−0.358***	0.603***	0.81

Diagonal values represent the square root of the average variance extracted (√AVE), and off-diagonal values represent latent construct correlations obtained from the five-factor CFA model. *p < 0.05. **p < 0.01. ***p < 0.001.

HTMT analysis indicated that all values were below the recommended threshold of 0.85 (Henseler et al., 2015). As shown in Table 5, HTMT values ranged from 0.140 to 0.660, with the highest value observed between psychological flexibility and posttraumatic growth (HTMT = 0.660). These findings support the discriminant validity of the constructs.

**Table 5:** Heterotrait–monotrait (HTMT) ratios.

Variable	1	2	3	4	5
1. Shame	-				
2. Guilt	0.360	-			
3. Fear	0.299	0.140	-		
4. Psychological flexibility	0.276	0.171	0.512	-	
5. Posttraumatic growth	0.166	0.153	0.366	0.660	-

### Assessment of potential common method variance

Multiple *post hoc* procedures were used to evaluate the potential influence of common method variance (CMV). First, Harman’s single-factor test indicated that the first unrotated factor accounted for 33.15% of the total variance. This result was considered descriptive and was not interpreted as evidence that common method bias was absent. Second, a single-factor confirmatory factor analysis using all 16 indicators demonstrated poor model fit, *χ*^2^(104) = 1762.42, *p* < 0.001, *χ*^2^/df = 16.95, CFI = 0.520, TLI = 0.446, and RMSEA = 0.201, 90% CI [0.193, 0.209], indicating that the covariance among the observed indicators was not adequately represented by a single general factor.

Finally, an equal-loading orthogonal unmeasured latent method construct (ULMC) model was estimated. The method factor was specified to load on all 16 indicators, with the method loadings constrained to equality, the method-factor variance fixed to 1, and the method factor specified as orthogonal to the substantive factors. This model did not meaningfully improve model fit relative to the original measurement model, Δ*χ*^2^ = 0.000, Δdf = 1, ΔCFI = −0.001, ΔTLI = −0.001, and ΔRMSEA = +0.001, and produced a higher AIC value, ΔAIC = +2.000. Standardized factor loadings also remained substantively similar across the original and ULMC models (see Table 6).

**Table 6:** Comparison of fit indices for the measurement model and the equal-loading ULMC model.

Model	*χ* ^2^	df	CFI	TLI	RMSEA	AIC
Measurement model (CFA)	223.146	94	0.963	0.952	0.059	307.146
Equal-loading ULMC model	223.146	93	0.962	0.951	0.060	309.146
Δ (difference)	0.000	1	−0.001	−0.001	0.001	+2.000

Δdf represents the absolute difference in degrees of freedom; changes in CFI, TLI, RMSEA, and AIC were calculated as the equal-loading ULMC model minus the measurement model.

Taken together, these analyses did not suggest that a single general method factor dominated the covariance structure. However, because all focal variables were assessed using self-report measures administered to the same participants at a single time point, these post hoc statistical procedures cannot rule out more complex forms of common method variance or determine whether shared method effects influenced the magnitude of the estimated associations.

### Structural model and model comparison

**Figure 1:**
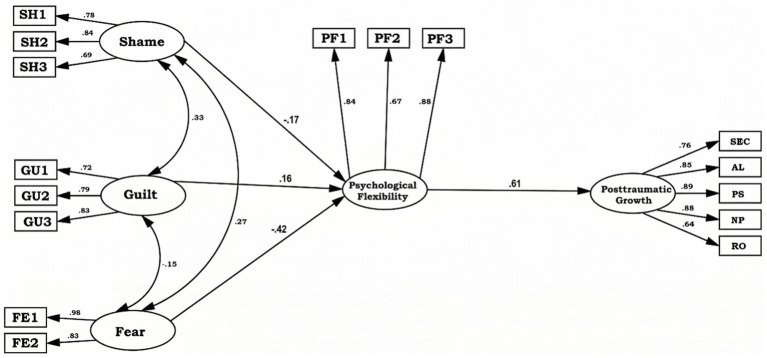
Structural equation model including measurement and structural components with standardized path coefficients. SH1–SH3, shame indicators; GU1–GU3, guilt indicators; FE1–FE2, fear indicators; PF1–PF3, psychological flexibility parcels; SEC, spiritual–existential change; AL, appreciation of life; PS, personal strength; NP, new possibilities; RO, relating to others.

The theoretical model was tested using Structural Equation Modeling (SEM), and both the measurement and structural components of the model are presented in Figure 1. Only standardized estimates are displayed in the figure.

Results for the constrained indirect-only model indicated acceptable model fit [*χ*^2^(97) = 226.54, *χ*^2^/df = 2.33, CFI = 0.963, TLI = 0.954, RMSEA = 0.058, AIC = 304.54]. The direct-and-indirect-paths model also demonstrated acceptable fit [*χ*^2^(94) = 223.15, *χ*^2^/df = 2.37, CFI = 0.963, TLI = 0.952, RMSEA = 0.059, AIC = 307.15].

To evaluate whether adding direct paths from guilt, shame, and fear to posttraumatic growth improved model fit, the indirect-only model was compared with the direct-and-indirect-paths model (see Table 7).

**Table 7:** Model comparison results.

Model	*χ* ^2^	df	*χ*^2^/df	CFI	TLI	RMSEA	AIC
Direct-and-indirect model	223.146	94	2.37	0.963	0.952	0.059	307.146
Constrained indirect-only model	226.540	97	2.33	0.963	0.954	0.058	304.540

The difference between the models was not statistically significant (Δ*χ*^2^ = 3.39, Δdf = 3, *p* > 0.05).

Chi-square difference testing indicated that adding the direct paths did not significantly improve model fit, Δ*χ*^2^ = 3.39, Δdf = 3, *p* > 0.05. The indirect-only model was therefore retained because it had a lower Akaike Information Criterion value and was more parsimonious. In the retained model, guilt, shame, and fear showed statistical indirect associations with posttraumatic growth through psychological flexibility.

### Structural model results

Standardized path coefficients in the retained indirect-only model indicated that shame (*β* = −0.170, *p* = 0.007) and fear (*β* = −0.423, *p* < 0.001) were negatively associated with psychological flexibility, whereas guilt was positively associated with psychological flexibility (*β* = 0.158, *p* = 0.009). The estimated associations differed in direction and magnitude across the emotional variables. Furthermore, psychological flexibility was positively associated with posttraumatic growth (*β* = 0.613, *p*  < 0.001).

Regarding model-based explained variance, shame, guilt, and fear jointly accounted for 27.3% of the variance in psychological flexibility (*R*^2^ = 0.273), whereas psychological flexibility accounted for 37.6% of the variance in posttraumatic growth within the specified model (*R*^2^ = 0.376; see Table 8).

**Table 8:** Standardized path coefficients and model-based explained variance for the retained indirect-only model.

Endogenous variable	Predictor	*β*	*p*	*R^2^*
Psychological flexibility	Shame	−0.170	0.007	0.273
	Guilt	0.158	0.009	
	Fear	−0.423	< 0.001	
Posttraumatic growth	Psychological flexibility	0.613	< 0.001	0.376

*β* = standardized path coefficient; *R*^2^ = proportion of variance accounted for within the specified model.

### Bootstrap analysis of statistical indirect effects

Bootstrapping with 5,000 resamples and 95% bias-corrected confidence intervals was used to assess the significance of the estimated statistical indirect effects.

The bootstrapped results indicated statistically significant indirect associations of fear, shame, and guilt with posttraumatic growth through psychological flexibility. The standardized indirect association for fear was negative, *β*__indirect_ = −0.259, 95% bias-corrected CI [−0.343, −0.187], *p* < 0.001. The standardized indirect association for shame was also negative, *β*__indirect_ = −0.105, 95% bias-corrected CI [−0.188, −0.030], *p* = 0.007.

In contrast, guilt showed a statistically significant positive standardized indirect association, *β*__indirect_ = 0.097, 95% bias-corrected CI [0.021, 0.180], *p* = 0.011.

As shown in Table 9, none of the confidence intervals included zero, indicating that all estimated statistical indirect effects were statistically significant. These findings were consistent with the retained cross-sectional indirect-effects model. Because all variables were measured concurrently, the estimated indirect effects should not be interpreted as evidence of temporal precedence, causal mediation, or a psychological mechanism.

**Table 9:** Bootstrapped indirect associations through psychological flexibility.

Model-implied indirect path	β_indirect_	95% CI
Fear → psychological flexibility → posttraumatic growth	−0.259	[−0.343, −0.187]
Shame → psychological flexibility → posttraumatic growth	−0.105	[−0.188, −0.030]
Guilt → psychological flexibility → posttraumatic growth	0.097	[0.021, 0.180]

Indirect associations were estimated using 5,000 bootstrap resamples and 95% bias-corrected confidence intervals. An indirect association was considered statistically significant when its confidence interval did not include zero. These cross-sectional indirect associations do not establish temporal ordering or causal mediation.

### Sensitivity analyses of the parcel-based specification

Two sensitivity analyses were conducted to examine whether the parcel-based representation obscured measurement heterogeneity or materially influenced the structural findings.

First, psychological flexibility was represented using its five original subscale scores. This subscale-indicator model demonstrated acceptable global fit, *χ*^2^(128) = 286.98, *χ*^2^/df = 2.24, CFI = 0.951, TLI = 0.942, and RMSEA = 0.056, 90% CI [0.047, 0.065]. However, the standardized subscale loadings were heterogeneous: values-based action = 0.83, present-moment awareness = 0.16, acceptance = −0.04, self-as-context = 0.55, and cognitive defusion = 0.59. Thus, acceptable global model fit coexisted with substantial local measurement heterogeneity, particularly for present-moment awareness and acceptance.

Second, the hypothesized indirect-effects model was re-estimated without parcel indicators using the observed total scores of shame, guilt, fear, psychological flexibility, and posttraumatic growth. The non-parceled model demonstrated good fit, *χ*^2^(3) = 6.67, *p* = 0.083, *χ*^2^/df = 2.22, CFI = 0.988, TLI = 0.962, and RMSEA = 0.056, 90% CI [0.000, 0.114], PCLOSE = 0.355. Shame was negatively associated with psychological flexibility, B = −0.357, SE = 0.101, *β* = −0.172, *p* < 0.001; guilt was positively associated with psychological flexibility, B = 0.378, SE = 0.124, *β* = 0.145, *p* = 0.002; and fear was negatively associated with psychological flexibility, B = −0.749, SE = 0.093, *β* = −0.376, *p* < 0.001. Psychological flexibility was positively associated with posttraumatic growth, B = 0.747, SE = 0.057, *β* = 0.550, *p* < 0.001. The model accounted for 22.3% of the variance in psychological flexibility and 30.2% of the variance in posttraumatic growth.

The standardized indirect associations also remained statistically significant in the non-parceled model: fear, *β* = −0.207, 95% bias-corrected CI [−0.270, −0.152], *p* < 0.001; shame, *β* = −0.095, 95% bias-corrected CI [−0.151, −0.045], *p* = 0.001; and guilt, *β* = 0.080, 95% bias-corrected CI [0.025, 0.139], *p* = 0.005. Accordingly, the directions and statistical significance of the principal structural and indirect associations were retained when the model was estimated without parcel indicators. Nevertheless, the subscale-indicator analysis showed that parceling attenuated meaningful facet-level heterogeneity and should not be interpreted as evidence that the five psychological flexibility dimensions form a homogeneous higher-order construct.

## Discussion

The present study examined indirect associations among guilt, shame, fear, psychological flexibility, and posttraumatic growth in a predominantly female Turkish convenience sample of adults who reported having experienced dating violence. Shame and fear were negatively associated with psychological flexibility, whereas guilt was positively associated with psychological flexibility; psychological flexibility, in turn, was positively associated with posttraumatic growth. These findings should be interpreted as associations among concurrently measured psychological variables within an exposure-selected sample. The model did not estimate the effect of dating violence itself and cannot determine whether the observed associations differ according to the severity, frequency, duration, recency, or type of violence. Moreover, because women constituted the substantial majority of the sample, the findings primarily reflect patterns observed among female participants and should not be interpreted as evidence that the same structural relationships necessarily apply to men or other gender groups.

Consistent with theoretical perspectives distinguishing self-evaluative processes from environmental threat detection (Tracy and Robins, 2004, 2007), guilt, shame, and fear were conceptualized as functionally distinct emotional experiences grounded in different psychological systems. This approach is consistent with evidence indicating that posttraumatic growth is not uniformly reported following trauma exposure and may be associated with how individuals relate to accompanying emotional experiences. Although posttraumatic growth has been documented in the context of intimate partner violence (Ceylan and Kışlak, 2024), IPV exposure is also associated with depression, posttraumatic stress disorder, and suicidality (White et al., 2024). Accordingly, reports of growth should not be interpreted as indicating the absence of psychological distress (Tedeschi et al., 2018).

The findings further identified statistical indirect associations between guilt, shame, fear, and posttraumatic growth through psychological flexibility. Psychological flexibility is commonly conceptualized as a broad, multidimensional regulatory construct concerning how individuals relate to distressing internal experiences rather than merely as an indicator of adjustment (Gloster et al., 2017; Hayes et al., 2006; Kashdan and Rottenberg, 2010). It involves openness to internal experiences and the capacity to engage in behavior consistent with personally meaningful values without requiring the suppression or elimination of difficult thoughts and emotions. Psychological flexibility may therefore represent a theoretically relevant correlate of the conditions under which posttraumatic growth is reported. However, the present cross-sectional findings do not establish that psychological flexibility operates as a causal mechanism or temporally precedes posttraumatic growth.

Gender may represent a potential source of heterogeneity in the observed associations. Experiences of dating violence, the meanings attributed to victimization, and emotional responses such as guilt, shame, and fear may be shaped by gendered social expectations and by differences in the type, severity, and relational context of violence. Psychological flexibility and perceived posttraumatic growth may likewise operate differently across gender groups. Because the present sample included only 59 male participants and did not assess gender-specific patterns of violence exposure, the study could not determine whether the measurement structure or structural pathways were equivalent across gender groups. The pooled coefficients should therefore be understood as estimates derived predominantly from female participants rather than as gender-invariant parameters.

### Differentiated association patterns across emotional constructs

Within this predominantly female sample, shame and fear were negatively associated with psychological flexibility, whereas guilt showed a positive association. These results highlight the value of distinguishing emotional experiences according to their theoretical functions rather than treating them as equivalent forms of negative affect.

Fear showed the largest absolute standardized association with psychological flexibility among the three emotional variables. This pattern is consistent with theoretical accounts involving threat-focused attention and restricted contextual responsiveness. According to Attentional Control Theory, anxiety and threat-related processing may reduce the efficiency of goal-directed attentional control and increase the influence of stimulus-driven attention (Eysenck and Derakshan, 2011). The observed association may therefore be compatible with rigid and threat-focused processing. However, attentional control, cognitive constriction, and experiential avoidance were not directly measured, and this explanatory account cannot be established from the present data. The finding indicates an association between fear and lower psychological flexibility rather than evidence that fear causally reduced psychological flexibility.

The negative association between shame and psychological flexibility is consistent with theoretical accounts linking shame with withdrawal and avoidance tendencies (Tangney et al., 2007). Shame has also been associated with greater posttraumatic stress symptoms (Shi et al., 2021). However, interpreting this relationship solely at the individual level may overlook potentially relevant relational and sociocultural influences. Shame may be associated with social evaluation, perceived stigma, interpersonal expectations, and concerns regarding relational acceptance (Mesquita and Walker, 2003; Tracy and Robins, 2004). Because collectivism, relational self-construal, perceived social evaluation, and culturally specific emotional norms were not directly assessed, the present findings cannot demonstrate that sociocultural factors shaped the observed association. These factors should instead be investigated as potential contextual variables in future research.

Importantly, shame retained a statistically significant negative association with psychological flexibility when fear and guilt were included simultaneously in the model. This pattern suggests that the association involving shame was not fully attributable to shared variance with threat-related fear or general self-conscious affect. From feminist and relational-trauma perspectives, shame following dating violence may reflect disruptions extending beyond immediate threat responses, including damage to relational trust, social identity, belongingness, and perceived self-worth. Repeated degradation, coercive control, victim-blaming, and perceived social devaluation may contribute to a global sense of being unacceptable, damaged, or disconnected from others, which may in turn be associated with greater experiential avoidance and reduced openness to difficult internal experiences. Contemporary models of posttraumatic growth similarly emphasize the reconstruction of identity, meaning, relational security, and connection following interpersonal trauma. Qualitative research with women who had experienced intimate partner violence similarly identified shame, diminished self-worth, insecurity, isolation, mixed negative emotions, and perpetrator- and system-related stressors as obstacles to perceived growth (Bryngeirsdottir and Halldorsdottir, 2022a). This qualitative convergence provides a relationally grounded context for the present shame and fear associations; however, because the current measures assessed general emotional tendencies rather than violence-specific emotions, it does not establish that participants’ shame or fear resulted from their dating-violence experiences. The observed shame–psychological flexibility association may therefore be compatible with the possibility that relationally produced shame is linked to difficulties engaging in the identity-, meaning-, and relationship-reconstruction processes associated with self-reported growth. However, relational trust, belongingness, social identity, self-worth, coercive control, and victim-blaming were not directly measured, and the present cross-sectional findings cannot establish that these processes account for the observed association. Moreover, because formal comparisons among the structural coefficients were not conducted, the shame pathway should be interpreted as a distinct statistically significant association rather than as demonstrably stronger than the other emotional pathways.

### Guilt: adaptive potential and its limits

A small but statistically significant positive association was observed between guilt and psychological flexibility, and guilt also showed a statistically significant positive indirect association with posttraumatic growth through psychological flexibility. Although the guilt-related indirect association was statistically significant, its standardized magnitude was small. Therefore, this finding should be interpreted as a modest correlational pattern rather than as evidence of substantial theoretical or clinical importance. From an ACT-consistent perspective, behavior-focused guilt could, under some conditions, coexist with responsibility-taking and value-consistent action. However, this interpretation remains tentative because the present study assessed general guilt rather than trauma-specific or functionally differentiated forms of guilt. Consistent with this caution, prior research has linked guilt to posttraumatic stress, particularly when guilt takes ruminative or self-punitive forms (Shi et al., 2021; Tangney et al., 2007).

The measure used in the present study assessed general guilt and did not distinguish behavior-focused, adaptive, maladaptive, ruminative, survivor, trauma-related, or victimization-related guilt. It also did not establish whether participants’ guilt was specifically related to their dating-violence experiences. The positive association should therefore not be interpreted as evidence that guilt promoted psychological flexibility, functioned adaptively, or encouraged value-consistent action. It indicates only that higher general guilt scores were associated with higher psychological flexibility scores in this sample. Future studies should use multidimensional and trauma-specific measures to determine whether theoretically distinct forms of guilt show different associations with psychological flexibility and posttraumatic growth.

### Patterns of statistical indirect associations in the structural model

Bootstrapped analyses indicated statistically significant negative indirect associations with posttraumatic growth through psychological flexibility for fear and shame, whereas guilt showed a statistically significant positive indirect association. These estimates represent statistical indirect associations among variables measured at the same time. They should not be interpreted as evidence that the emotional variables temporally preceded psychological flexibility or that psychological flexibility subsequently produced changes in posttraumatic growth.

The standardized structural coefficients showed that fear had the largest absolute association with psychological flexibility, whereas shame showed a smaller negative association and guilt showed a small positive association. These coefficients describe the strength and direction of the estimated associations between each emotional variable and psychological flexibility. They do not demonstrate that fear exerted a stronger causal influence than shame or guilt. Moreover, formal statistical comparisons between the path coefficients or indirect effects were not conducted. The estimated relationships should therefore be described as differing descriptively rather than statistically in direction and magnitude.

The contrasting directions of the associations may reflect the heterogeneous theoretical functions assigned to the three emotions. Fear is generally organized around threat detection, shame around global negative self-evaluation, and guilt around evaluations of specific actions or responsibilities. Nevertheless, the study did not directly test the proposed emotional functions or the specific psychological processes through which these emotions may be associated with psychological flexibility. The findings support differentiated association patterns but not distinct causal pathways.

### Psychological flexibility and posttraumatic growth

The positive association between psychological flexibility (PF) and posttraumatic growth (PTG) indicates that participants reporting greater psychological flexibility also reported greater posttraumatic growth. This pattern is consistent with theoretical frameworks associating PTG with cognitive restructuring and meaning-making (Tedeschi and Calhoun, 2004). Prior conceptual and empirical work has associated greater psychological flexibility with better mental health, well-being, and adaptive functioning (Gloster et al., 2017; Kashdan and Rottenberg, 2010), while recent empirical studies have specifically linked psychological flexibility with posttraumatic growth (Sarıkoç and Uğur, 2025). Nevertheless, the present cross-sectional association does not demonstrate that psychological flexibility facilitated, produced, or preceded psychological change.

At the same time, ongoing debates question whether PTG reflects genuine transformation or represents defensive cognitive reframing (Jayawickreme et al., 2021). Psychological flexibility involves experiential openness, contextual responsiveness, and value-consistent action (Kashdan and Rottenberg, 2010), and prior research has associated flexibility-related processes, such as cognitive flexibility and self-compassion, with PTG (Gudek Akpinar and Akis, 2025). However, the present data cannot determine whether the association between psychological flexibility and posttraumatic growth reflects enduring functional adaptation, positive reinterpretation, defensive reconstruction, reciprocal relationships, or their combination.

Accordingly, the findings support only the conclusion that psychological flexibility and self-reported posttraumatic growth were positively associated in the present sample. They do not establish that the reported growth was objectively verified, behaviorally manifested, or stable over time. Longitudinal studies incorporating repeated assessments, behavioral indicators, and informant reports would be required to evaluate whether reported posttraumatic growth corresponds to enduring psychological change and whether psychological flexibility temporally precedes such change.

### Interpreting the findings: theoretical and alternative perspectives

Examining psychological flexibility as a central statistical correlate is theoretically grounded in accounts of the relationship between emotional experience and behavioral regulation (Kashdan and Rottenberg, 2010). However, alternative explanations should be considered. Psychological flexibility and posttraumatic growth may be reciprocally related, posttraumatic growth may be associated with subsequent increases in psychological flexibility, or both constructs may be influenced by third variables such as general negative affect, depressive symptoms, posttraumatic stress symptoms, trauma severity, social support, perceived safety, coping, meaning-making, or the time elapsed since the most recent or most salient dating-violence experience. These variables were not directly assessed and therefore could not be incorporated into the structural model as covariates. Consequently, the present analyses cannot determine whether psychological flexibility accounts for unique variance in posttraumatic growth beyond these established psychological, interpersonal, and trauma-related correlates. Their omission may also have influenced the magnitude of the estimated structural coefficients and statistical indirect associations. Because the study used cross-sectional self-report data, the proposed ordering cannot be empirically distinguished from these alternative models. Accordingly, the proposed model should be regarded as one theoretically plausible correlational representation of the observed associations rather than as a uniquely supported explanatory model.

The *post hoc* common method variance analyses did not suggest that a single general method factor adequately accounted for the observed covariance among the study variables. Nevertheless, all focal constructs were assessed through self-report measures administered to the same participants at the same time point. Consequently, shared response tendencies, social desirability, consistency motives, item-context effects, or other sources of common method variance may have influenced the magnitude of the observed correlations and indirect associations. Harman’s single-factor test, single-factor CFA, and ULMC modeling cannot eliminate these possibilities. The structural model should therefore be interpreted as a correlational representation of how the concurrently measured variables covaried rather than as method-independent evidence of the proposed associations, their temporal ordering, or an underlying psychological mechanism.

The study contributes to the literature in three principal ways: (1) identifying differentiated association patterns for self-conscious and threat-related emotions within the same structural model, (2) estimating the statistical indirect associations of guilt, shame, and fear with posttraumatic growth through psychological flexibility, and (3) demonstrating that the directions and statistical significance of the principal associations were retained across both the parcel-based latent-variable model and the observed-total-score sensitivity model, despite the measurement heterogeneity identified at the psychological-flexibility subscale level.

### Clinical implications

The clinical implications of these findings should be considered preliminary. Because dating-violence characteristics were not measured, psychological flexibility was assessed as a broad construct, and the data were cross-sectional, the results do not establish that interventions targeting psychological flexibility will produce posttraumatic growth specifically in response to dating violence. These findings suggest that psychological flexibility may be a clinically relevant construct to consider in populations reporting dating-violence exposure; however, longitudinal and intervention-based studies are needed before drawing conclusions about directionality, therapeutic efficacy, or posttraumatic growth specifically attributable to dating-violence recovery.

More broadly, trauma-informed intervention development may need to situate psychological flexibility within the relational and ecological context of recovery. Clinical assessment and intervention planning may need to consider current safety, continued contact with the person who used violence, ongoing coercive control, social support, practical and economic stressors, and legal or institutional barriers alongside individuals’ relationships with difficult internal experiences. These contextual domains were not assessed in the present study and therefore cannot be inferred from the current model; however, qualitative and theory-synthesis work has identified them as potentially relevant facilitators or barriers to recovery and perceived growth following intimate partner violence (Bryngeirsdottir and Halldorsdottir, 2022a; Bryngeirsdottir et al., 2022).

For clinicians working with populations resembling the predominantly female sample examined here, individuals’ relationships with difficult emotional experiences may warrant cautious consideration alongside symptom-focused approaches. However, the cross-sectional design and gender imbalance do not establish that the same intervention priorities apply equally across gender groups. The observed associations suggest that future intervention studies could examine whether supporting more flexible responses to fear- and shame-related experiences, together with value-consistent behavior, is associated with improved prospective outcomes. Nevertheless, shame-related experiential avoidance was not directly measured, and the study did not test cognitive defusion, self-as-context, acceptance, or other ACT components as distinct therapeutic mechanisms. Therefore, no specific ACT technique can be recommended on the basis of the present findings alone.

Given the comparatively strong association between fear and psychological flexibility in this sample, future longitudinal and intervention studies could examine the processes underlying this association and whether changes in these processes are related to prospective clinical outcomes. Replication in gender-balanced samples is needed before broader clinical implications can be considered.

The positive association involving guilt should also be interpreted cautiously. The guilt measure assessed general guilt and did not distinguish adaptive, maladaptive, behavioral, survivor, trauma-related, or self-blaming forms of guilt, nor did it establish whether participants’ guilt was specifically related to their dating-violence experiences. Accordingly, the finding should not be interpreted as evidence that guilt functions as an adaptive signal or facilitates value-consistent action. Given the small indirect coefficient and the lack of guilt-type differentiation, future clinical research should use multidimensional and trauma-specific guilt measures before deriving intervention recommendations concerning guilt.

### Conceptual implications for posttraumatic growth

The findings suggest that posttraumatic growth may be considered alongside symptom reduction as a potentially relevant outcome in future longitudinal and intervention research. The present cross-sectional findings do not establish that posttraumatic growth can be directly produced through changes in psychological flexibility.

Trauma-specific accounts of posttraumatic growth following intimate partner violence further suggest that perceived growth may coexist with triggers, continuing distress, relational difficulties, and other lingering consequences of violence, and may fluctuate rather than represent a permanent endpoint (Bryngeirsdottir et al., 2022). Accordingly, higher posttraumatic growth scores should not be interpreted as evidence that fear, shame, psychological distress, safety concerns, or the practical consequences of violence have resolved. Future longitudinal research should assess posttraumatic growth alongside ongoing distress, perceived safety, relational functioning, and changes in exposure circumstances.

### Limitations

Several limitations warrant caution in interpreting these results. First, the cross-sectional design precludes causal inference, the establishment of temporal precedence, and conclusions regarding the direction or temporal dynamics of the observed associations. Psychological flexibility and posttraumatic growth may be reciprocally related, or the observed relationships may reflect alternative temporal orderings that cannot be distinguished using concurrently collected data.

Second, all focal variables were assessed through self-report measures administered to the same participants at a single time point, leaving a substantial concern regarding common method variance. Although Harman’s single-factor test, single-factor CFA, and ULMC modeling did not suggest that a single general method factor dominated the covariance structure, these *post hoc* procedures cannot rule out more complex forms of common method bias. Shared response styles, social desirability, consistency tendencies, item-context effects, and the common measurement context may therefore have influenced the magnitude of the observed correlations and statistical indirect associations. Future studies should reduce this risk through temporal separation of predictor and outcome assessments, multiple informants or data sources, clinician-rated or behavioral measures, and prospectively selected marker variables.

Third, several theoretically relevant alternative explanatory variables were not assessed. The study did not measure or statistically control for general negative affectivity, depressive symptoms, posttraumatic stress symptoms, social support, perceived safety, coping, trauma severity, or the time elapsed since the most recent or most salient dating-violence experience. These factors may be associated with guilt, shame, fear, psychological flexibility, and self-reported posttraumatic growth and may therefore account for part of the observed relationships. Because these variables could not be incorporated into the structural model as covariates, omitted-variable confounding cannot be ruled out. Accordingly, the findings do not establish that psychological flexibility accounts for unique variance in posttraumatic growth beyond these established psychological, interpersonal, and trauma-related correlates, and confidence in the specificity of the proposed model is limited.

Fourth, dating-violence exposure was assessed using a single unvalidated dichotomous self-report item and served solely as an eligibility and sample-defining criterion. Because all participants in the analytical sample endorsed this item, dating-violence status showed no between-participant variability and could not be incorporated into the structural model. The study also did not assess the form, severity, frequency, duration, recency, chronicity, cumulative burden, relational context, or specific type of violence using a validated multidimensional measure. The study also did not determine whether the violent dating relationship had ended, whether participants remained in contact with the person who used violence, or whether ongoing threat, coercive control, stalking, or post-separation stressors were present. These distinctions are important because fear, psychological flexibility, and self-reported posttraumatic growth may have different meanings under conditions of continuing danger versus post-relationship recovery. Consequently, the findings cannot determine whether the observed associations vary according to exposure burden, time since exposure, or psychological, physical, sexual, digital, and controlling forms of dating violence. The results should therefore be interpreted as associations among emotional experiences, psychological flexibility, and posttraumatic growth within a self-reported dating-violence sample rather than as evidence regarding the psychological effects of dating violence itself.

Fifth, the sampling strategy and gender composition limit the generalizability of the findings. Participants were recruited through convenience sampling, and the sample was predominantly female, comprising 335 participants who selected “Female,” 59 who selected “Male,” and two who selected “Other.” The structural estimates were therefore derived predominantly from data provided by female participants and should not be assumed to generalize to male participants or other gender groups. Gender may be associated with the experience and interpretation of dating violence, the type and severity of violence exposure, emotional responses such as guilt, shame, and fear, psychological flexibility, and perceived posttraumatic growth. The comparatively small number of male participants, together with the very small number of participants who selected “Other,” did not permit adequately powered measurement-invariance analyses or stable multigroup comparisons of the structural pathways. Accordingly, the findings are most appropriately generalized to predominantly female convenience samples with characteristics similar to those included in the present study.

Sixth, the guilt measure assessed general guilt and did not distinguish adaptive, maladaptive, behavior-focused, ruminative, survivor, trauma-related, or victimization-related forms of guilt. It also did not establish whether participants’ guilt was specifically related to their dating-violence experiences. Consequently, the positive association between guilt and psychological flexibility cannot be interpreted as evidence that trauma-related or victimization-related guilt is adaptive or that guilt promotes psychological flexibility.

Seventh, the psychometric suitability of the Guilt–Shame Scale for dating-violence and intimate partner violence populations remains uncertain. The item-level CFA provided only partial support for the proposed two-factor structure: although *χ*^2^/df, RMSEA, and GFI indicated acceptable fit, CFI, TLI, and IFI remained below conventional thresholds. The guilt and shame estimates should therefore be interpreted with additional caution and should not be regarded as evidence that the measure has been fully validated for use in these populations.

Eighth, although guilt, shame, and fear were conceptually distinguished as functionally different emotional systems, these functional distinctions were not directly tested at the measurement level. Similarly, psychological flexibility was operationalized as a broad latent construct, and the study did not examine acceptance, cognitive defusion, present-moment awareness, self-as-context, or values-based action as distinct process-specific mechanisms. The findings therefore support differentiated patterns of association among broad constructs but do not establish distinct emotional or ACT-based mechanisms.

Ninth, the measurement of psychological flexibility presented additional psychometric limitations. The cognitive-defusion dimension demonstrated low internal consistency (*α* = 0.55), and the sensitivity model using the five original subscale scores revealed substantial heterogeneity in the extent to which the dimensions represented the broader psychological flexibility construct. Although the non-parceled observed-score sensitivity analysis reproduced the directions and statistical significance of the principal structural and indirect associations, item parceling may have attenuated facet-level measurement heterogeneity. The psychological flexibility findings should therefore be interpreted at the level of an aggregated broad score and should not be treated as evidence that the five dimensions form a homogeneous higher-order construct or generalized to any specific ACT process.

Tenth, posttraumatic growth was assessed exclusively through self-report. It was therefore not possible to determine whether higher scores reflected enduring psychological or behavioral transformation, adaptive meaning reconstruction, positive reinterpretation, or defensively constructed perceptions of growth (Jayawickreme et al., 2021). Longitudinal assessments, behavioral indicators, and informant reports would be required to evaluate the stability and external validity of reported posttraumatic growth.

Finally, the study was conducted in Türkiye, but cultural constructs such as collectivism, relational self-construal, perceived social expectations, victim-blaming norms, and culturally specific meanings of guilt, shame, fear, and posttraumatic growth were not directly measured. The sociogeographic context of the study therefore does not provide empirical support for culture-specific explanations of the findings. Direct measurement of cultural variables, cross-cultural comparisons, and measurement-invariance analyses would be required to evaluate whether the observed associations differ across cultural contexts.

Despite these limitations, the study offers a theoretically grounded correlational model identifying differentiated associations among guilt, shame, fear, psychological flexibility, and self-reported posttraumatic growth. The findings provide a basis for future longitudinal, multi-method, and intervention-based research designed to evaluate temporal ordering, alternative explanations, measurement robustness, and the specificity of the proposed relationships.

### Recommendations

Future research should employ longitudinal, cross-lagged, and experimental designs to evaluate temporal ordering and reciprocal associations between psychological flexibility and posttraumatic growth. Theoretically, studies should move beyond valence-based classifications toward integrative models that differentiate emotional experiences according to their functional and cognitive characteristics. Measurement-level refinements, including the use of multidimensional and trauma-specific measures of guilt, shame, and fear, would permit more precise examination of how distinct emotional forms are associated with psychological flexibility and posttraumatic growth.

Future studies should use validated multidimensional measures of dating violence that distinguish psychological, physical, sexual, digital, and controlling behaviors and assess their frequency, severity, duration, and recency. Incorporating these exposure characteristics into longitudinal structural models would allow researchers to examine whether the observed psychological associations vary across patterns and levels of violence exposure and whether changes in psychological flexibility temporally precede, follow, or reciprocally relate to changes in posttraumatic growth.

From an applied perspective, prospective and randomized intervention studies are needed to test whether treatment-related changes in psychological flexibility are followed by changes in posttraumatic growth, psychological distress, perceived safety, and relational functioning. Future research should also directly assess cultural variables, such as social approval, normative pressure, relational self-construal, and victim-blaming norms, rather than inferring cultural processes from the sociogeographic setting of the sample. Finally, multi-informant, longitudinal, and behaviorally anchored designs are needed to distinguish enduring psychological change from coping-related positive appraisal or reconstructed perceptions of growth.

## Conclusion

In this predominantly female Turkish convenience sample of adults who reported having experienced dating violence, guilt, shame, and fear showed differentiated cross-sectional indirect associations with posttraumatic growth through psychological flexibility. Shame and fear were negatively associated with psychological flexibility, whereas guilt was positively associated with psychological flexibility; psychological flexibility was positively associated with self-reported posttraumatic growth. These findings identify a differentiated correlational pattern among concurrently measured emotional experiences, psychological flexibility, and posttraumatic growth, with psychological flexibility representing a potentially relevant statistical linking variable within the specified model.

The findings concern associations among the measured psychological variables within an exposure-selected sample. Dating violence itself was not modeled as a predictor or explanatory construct, and the results therefore do not indicate how the severity, frequency, duration, recency, relational context, or type of dating violence relates to psychological flexibility or posttraumatic growth. Moreover, because the sample was recruited through convenience sampling and consisted predominantly of female participants, the observed structural relationships should not be assumed to operate equivalently among male participants or other gender groups. Replication using more representative and gender-balanced samples, together with formal measurement-invariance testing and multigroup analyses, is needed before broader conclusions can be drawn.

## Data Availability

The raw data supporting the conclusions of this article will be made available by the authors, without undue reservation.

## References

[ref1] Akcan G. Bakkal B. H. Elmas Ö. (2024). The mediation role of coping with stress in the relationship between psychological flexibility and posttraumatic growth in cancer patients. Omega. doi: 10.1177/00302228241292381 39394671

[ref2] Bakaitytė A. Kaniušonytė G. Truskauskaitė-Kunevičienė I. Žukauskienė R. (2022). Longitudinal investigation of posttraumatic growth in female survivors of intimate partner violence: the role of event centrality and identity exploration. J. Interpers. Violence 37:NP1058–NP1076. doi: 10.1177/0886260520920864, 32410496

[ref3] Baumeister R. F. Stillwell A. M. Heatherton T. F. (1994). Guilt: an interpersonal approach. Psychol. Bull. 115, 243–267. doi: 10.1037/0033-2909.115.2.243, 8165271

[ref4] Bruno F. Vozzo F. Arcuri D. Maressa R. La Cava E. Malvaso A. . (2024). The longitudinal association between perceived stress, PTSD symptoms, and post-traumatic growth during the COVID-19 pandemic: the role of coping strategies and psychological inflexibility. Curr. Psychol. 43, 13871–13886. doi: 10.1007/s12144-022-03502-3, 35910236 PMC9323876

[ref5] Bryngeirsdottir H. S. Arnault D. S. Halldorsdottir S. (2022). The post-traumatic growth journey of women who have survived intimate partner violence: a synthesized theory emphasizing obstacles and facilitating factors. Int. J. Environ. Res. Public Health 19:8653. doi: 10.3390/ijerph19148653, 35886504 PMC9321137

[ref6] Bryngeirsdottir H. S. Halldorsdottir S. (2022a). Fourteen main obstacles on the journey to post-traumatic growth as experienced by female survivors of intimate partner violence: “it was all so confusing.”. Int. J. Environ. Res. Public Health 19:5377. doi: 10.3390/ijerph19095377, 35564770 PMC9101378

[ref7] Bryngeirsdottir H. S. Halldorsdottir S. (2022b). “I’m a winner, not a victim”: the facilitating factors of post-traumatic growth among women who have suffered intimate partner violence. Int. J. Environ. Res. Public Health 19:1342. doi: 10.3390/ijerph19031342, 35162363 PMC8834824

[ref8] Ceylan M. Kışlak Ş. (2024). Posttraumatic growth in battered women and the reflection of violence on post-divorce growth: a systematic review. Psikiyatride Güncel Yaklaşımlar 16, 740–752. doi: 10.18863/pgy.1379675

[ref9] Cohen J. (1988). Statistical Power Analysis for the Behavioral Sciences. 2nd Edn Hillsdale, NJ: Lawrence Erlbaum Associates.

[ref10] Elderton A. Berry A. Chan C. (2017). A systematic review of posttraumatic growth in survivors of interpersonal violence in adulthood. Trauma Violence Abuse 18, 223–236. doi: 10.1177/1524838015611672, 26459504

[ref11] Eysenck M. W. Derakshan N. (2011). New perspectives in attentional control theory. Pers. Individ. Differ. 50, 955–960. doi: 10.1016/j.paid.2010.08.019

[ref12] Feiring C. Taska L. (2005). The persistence of shame following sexual abuse: a longitudinal look at risk and recovery. Child Maltreat. 10, 337–349. doi: 10.1177/1077559505276686, 16204736

[ref13] Francis A. W. Dawson D. L. Golijani-Moghaddam N. (2016). The development and validation of the comprehensive assessment of acceptance and commitment therapy processes (CompACT). J. Context. Behav. Sci. 5, 134–145. doi: 10.1016/j.jcbs.2016.05.003

[ref14] Freyd J. J. (1996). Betrayal Trauma: The Logic of Forgetting Childhood Abuse. Cambridge, MA: Harvard University Press.

[ref15] Gloster A. T. Meyer A. H. Lieb R. (2017). Psychological flexibility as a malleable public health target: evidence from a representative sample. J. Context. Behav. Sci. 6, 166–171. doi: 10.1016/j.jcbs.2017.02.003

[ref16] Gross J. J. (2015). Emotion regulation: current status and future prospects. Psychol. Inq. 26, 1–26. doi: 10.1080/1047840X.2014.940781

[ref17] Gudek Akpinar S. Akis A. D. (2025). The mediating role of cognitive flexibility and self-compassion in the relationship between complex trauma and posttraumatic growth. J. West Eur. Soc. Sci. 2, 246–258. doi: 10.5281/zenodo.15123078

[ref18] Hair J. F. Black W. C. Babin B. J. Anderson R. E. (2019). Multivariate Data Analysis. 8th Edn Andover: Cengage.

[ref19] Hayes S. C. Luoma J. B. Bond F. W. Masuda A. Lillis J. (2006). Acceptance and commitment therapy: model, processes and outcomes. Behav. Res. Ther. 44, 1–25. doi: 10.1016/j.brat.2005.06.006, 16300724

[ref20] Hayes S. C. Strosahl K. D. Wilson K. G. (1999). Acceptance and Commitment Therapy: An Experiential Approach to Behavior Change. New York, NY: Guilford Press.

[ref21] Hayes S. C. Strosahl K. D. Wilson K. G. (2011). Acceptance and Commitment Therapy: The Process and Practice of Mindful Change. 2nd Edn New York, NY: Guilford Press.

[ref22] Henseler J. Ringle C. M. Sarstedt M. (2015). A new criterion for assessing discriminant validity in variance-based structural equation modeling. J. Acad. Mark. Sci. 43, 115–135. doi: 10.1007/s11747-014-0403-8

[ref23] Herman J. L. (1992). Trauma and Recovery. New York, NY: Basic Books.

[ref24] Janoff-Bulman R. (1992). Shattered Assumptions: Towards a New Psychology of Trauma. New York, NY: Free Press.

[ref25] Jayawickreme E. Infurna F. J. Alajak K. Blackie L. E. R. Chopik W. J. Chung J. M. (2021). Post-traumatic growth as positive personality change: challenges, opportunities, and recommendations. J. Pers. 89, 145–165. doi: 10.1111/jopy.12591, 32897574 PMC8062071

[ref26] Johnson M. P. (2008). A Typology of Domestic Violence: Intimate Terrorism, Violent Resistance, and Situational Couple Violence. Boston, MA: Northeastern University Press.

[ref27] Joseph S. Linley P. A. (2005). Positive adjustment to threatening events: an organismic valuing theory of growth through adversity. Rev. Gen. Psychol. 9, 262–280. doi: 10.1037/1089-2680.9.3.262

[ref28] Karakuş S. Akbay S. E. (2020). Psikolojik esneklik ölçeği: Uyarlama, geçerlik ve güvenirlik çalışması [psychological flexibility scale: adaptation, validity, and reliability study]. Mersin Univ. Egit. Fak. Derg. 16, 32–43. doi: 10.17860/mersinefd.665406

[ref29] Kashdan T. B. Rottenberg J. (2010). Psychological flexibility as a fundamental aspect of health. Clin. Psychol. Rev. 30, 865–878. doi: 10.1016/j.cpr.2010.03.001, 21151705 PMC2998793

[ref30] Lagdon S. Armour C. Stringer M. (2014). Adult experience of mental health outcomes as a result of intimate partner violence victimisation: a systematic review. Eur. J. Psychotraumatol. 5:24794. doi: 10.3402/ejpt.v5.24794, 25279103 PMC4163751

[ref31] Landi G. Pakenham K. I. Mattioli E. Crocetti E. Agostini A. Grandi S. . (2022). Post-traumatic growth in people experiencing high post-traumatic stress during the COVID-19 pandemic: the protective role of psychological flexibility. J. Contextual Behav. Sci. 26, 44–55. doi: 10.1016/j.jcbs.2022.08.008, 36060527 PMC9420208

[ref32] LeDoux J. E. (2000). Emotion circuits in the brain. Annu. Rev. Neurosci. 23, 155–184. doi: 10.1146/annurev.neuro.23.1.155, 10845062

[ref33] Leith K. P. Baumeister R. F. (1998). Empathy, shame, guilt, and narratives of interpersonal conflicts: guilt-prone people are better at perspective taking. J. Pers. 66, 1–37. doi: 10.1111/1467-6494.00001

[ref34] Lewis M. (2008). “Self-conscious emotions,” in Handbook of Emotions, eds. Lewis M. Haviland-Jones J. M. Barrett L. F.. 3rd ed (New York, NY: Guilford Press), 742–756.

[ref35] Li Y. Liu L. Wu X. Wang W. (2025). Dual effects of self-compassion on posttraumatic stress symptoms and posttraumatic growth: the roles of trauma-related shame and guilt. J. Psychol. 159, 227–244. doi: 10.1080/00223980.2024.2397690, 39255419

[ref36] Little T. D. Cunningham W. A. Shahar G. Widaman K. F. (2002). To parcel or not to parcel: exploring the question, weighing the merits. Struct. Equ. Model. 9, 151–173. doi: 10.1207/S15328007SEM0902_1

[ref37] Markus H. R. Kitayama S. (1991). Culture and the self: implications for cognition, emotion, and motivation. Psychol. Rev. 98, 224–253. doi: 10.1037/0033-295X.98.2.224

[ref38] Matsunaga M. (2008). Item parceling in structural equation modeling: a primer. Commun. Methods Meas. 2, 260–293. doi: 10.1080/19312450802458935

[ref39] Mesquita B. Walker R. (2003). Cultural differences in emotions: a context for interpreting emotional experiences. Behav. Res. Ther. 41, 777–793. doi: 10.1016/S0005-7967(02)00189-4, 12781245

[ref40] Morrison A. P. (1989). Shame: The Underside of Narcissism. Hillsdale, NJ: Analytic Press.

[ref41] Park C. L. (2010). Making sense of the meaning literature: an integrative review of meaning making and its effects on adjustment to stressful life events. Psychol. Bull. 136, 257–301. doi: 10.1037/a0018301, 20192563

[ref42] Prati G. Pietrantoni L. (2009). Optimism, social support, and coping strategies as factors contributing to posttraumatic growth: a meta-analysis. J. Loss Trauma 14, 364–388. doi: 10.1080/15325020902724271

[ref43] Rhatigan D. L. Axsom D. K. (2006). Using the investment model to understand battered women’s commitment to abusive relationships. J. Fam. Violence 21, 153–162. doi: 10.1007/s10896-005-9013-z

[ref44] Sachdeva J. Shukla R. (2024). Exploring the mediating influence of psychological flexibility and self-compassion on the relationship between adverse childhood experiences and post-traumatic growth among young adults in India. Psychol. Rep. doi: 10.1177/00332941241302318 39606864

[ref45] Şahin N. H. Şahin N. (1992). Adolescent Guilt, Shame, and Depression in Relation to Sociotropy and Autonomy. Toronto, Ontario, Canada: World Congress of Cognitive Therapy.

[ref46] Salcioglu E. Urhan S. Pirinccioglu T. Aydin S. (2017). Anticipatory fear and helplessness predict PTSD and depression in domestic violence survivors. Psychol. Trauma Theory Res. Pract. Policy 9, 117–125. doi: 10.1037/tra0000200, 27710008

[ref47] Sarıkoç M. Uğur E. (2025). Post-traumatic growth, psychological inflexibility, and the mediating role of intolerance of uncertainty in adolescents affected by the Kahramanmaraş earthquake. Sci. Rep. 15:36179. doi: 10.1038/s41598-025-19935-2, 41102342 PMC12533121

[ref48] Shi C. Ren Z. Zhao C. Zhang T. Chan S. H. (2021). Shame, guilt, and posttraumatic stress symptoms: a three-level meta-analysis. J. Anxiety Disord. 82:102443. doi: 10.1016/j.janxdis.2021.102443, 34265540

[ref49] Stark E. (2007). Coercive Control: How Men Entrap Women in Personal Life. New York, NY: Oxford University Press.

[ref50] Szyfer Lipinsky A. Goldner L. Hadar D. Saint-Arnault D. (2025). Predicting recovery pathways in Jewish ultra-orthodox intimate partner violence survivors: a structural equation modeling approach. J. Interpers. Violence 40, 974–1000. doi: 10.1177/08862605241255738, 38819011

[ref51] Tabachnick B. G. Fidell L. S. (2013). Using Multivariate Statistics. 6th Edn Boston, MA: Pearson.

[ref52] Tangney J. P. Stuewig J. Mashek D. J. (2007). Moral emotions and moral behavior. Annu. Rev. Psychol. 58, 345–372. doi: 10.1146/annurev.psych.56.091103.070145, 16953797 PMC3083636

[ref53] Tedeschi R. G. Calhoun L. G. (1996). The posttraumatic growth inventory: measuring the positive legacy of trauma. J. Trauma. Stress. 9, 455–471. doi: 10.1007/BF02103658, 8827649

[ref54] Tedeschi R. G. Calhoun L. G. (2004). Posttraumatic growth: conceptual foundations and empirical evidence. Psychol. Inq. 15, 1–18. doi: 10.1207/s15327965pli1501_01

[ref55] Tedeschi R. G. Cann A. Taku K. Şenol-Durak E. Calhoun L. G. (2017). The posttraumatic growth inventory: a revision integrating existential and spiritual change. J. Trauma. Stress. 30, 11–18. doi: 10.1002/jts.22155, 28099764

[ref56] Tedeschi R. G. Shakespeare-Finch J. Taku K. (2018). Posttraumatic Growth: Theory, Research, and Applications. New York, NY: Routledge.

[ref57] Tracy J. L. Robins R. W. (2004). Putting the self into self-conscious emotions: a theoretical model. Psychol. Inq. 15, 103–125. doi: 10.1207/s15327965pli1502_01

[ref58] Tracy J. L. Robins R. W. (2007). “The self in self-conscious emotions: a cognitive appraisal approach,” in The Self-Conscious Emotions: Theory and Research, (New York, NY: Guilford Press), 3–20.

[ref59] Türk F. (2024). Traumatic stress and post-traumatic growth in individuals who have had COVID-19: the mediating effect of resilience and moderating effect of psychological flexibility. PLoS One 19:e0310495. doi: 10.1371/journal.pone.0310495, 39774492 PMC11684618

[ref60] Turkish Statistical Institute (2025). Türkiye violence against women survey, 2024. Available online at: https://veriportali.tuik.gov.tr/en/press/57940 (Accessed July 24, 2026).

[ref61] Walker L. E. (1979). The Battered Woman. New York, NY: Harper & Row.

[ref62] Wang W. Wu X. Tian Y. (2018). Mediating roles of gratitude and social support in the relation between survivor guilt and posttraumatic stress disorder, posttraumatic growth among adolescents after the ya’an earthquake. Front. Psychol. 9:2131. doi: 10.3389/fpsyg.2018.02131, 30455660 PMC6230928

[ref63] White S. J. Sin J. Sweeney A. Salisbury T. Wahlich C. Montesinos Guevara C. M. . (2024). Global prevalence and mental health outcomes of intimate partner violence among women: a systematic review and meta-analysis. Trauma Violence Abuse 25, 494–511. doi: 10.1177/15248380231155529, 36825800 PMC10666489

[ref64] World Health Organization (2024). Violence against women. Available online at: https://www.who.int/news-room/fact-sheets/detail/violence-against-women (Accessed July 24, 2026.)

[ref65] Wu X. Kaminga A. C. Dai W. Deng J. Wang Z. Pan X. . (2019). The prevalence of moderate-to-high posttraumatic growth: a systematic review and meta-analysis. J. Affect. Disord. 243, 408–415. doi: 10.1016/j.jad.2018.09.023, 30268956

